# Evaluation of the antinociceptive activities of enaminone compounds on the formalin and hot plate tests in mice

**DOI:** 10.1038/srep21582

**Published:** 2016-02-26

**Authors:** Willias Masocha, Samuel B. Kombian, Ivan O. Edafiogho

**Affiliations:** 1 Department of Pharmacology and Therapeutics, Faculty of Pharmacy, Kuwait University, Kuwait; 2 Department of Pharmaceutical Sciences, School of Pharmacy, University of Saint Joseph, Hartford, CT 06103, USA

## Abstract

Recently, we found that methyl 4-(4′-bromophenyl)aminocyclohex-3-en-6-methyl-2-oxo-1-oate (E139), an anticonvulsant enaminone, has antinociceptive activity in the hot plate test. In this study we evaluated the antinociceptive activity of five anilino enaminones E139, ethyl 4-(4′-chlorophenyl)amino-6-methyl-2-oxocyclohex-3-en-1-oate (E121), ethyl 4-(4′-bromophenyl)amino-6-methyl-2-oxocyclohex-3-en-1-oate (E122), methyl 4-(4′-chlorophenyl)amino-6-methyl-2-oxocyclohex-3-en-1-oate (E138) and ethyl 4-(4′-fluorophenyl)amino-6-methyl-2-oxocyclohex-3-en-1-oate (BRG 19) using the formalin and hot plate tests. E139 has been reported to exert its effects via enhancement of extracellular GABA levels, thus tiagabine, a GABA transporter inhibitor, was evaluated as a control together with indomethacin. Tiagabine had antinociceptive activity in both phase 1 (neurogenic pain) and phase 2 (inflammatory pain) of the formalin test, whereas indomethacin had activity only in phase 2. E139 and E138 had antinociceptive activity in both phases of the formalin test, whereas E121 had activity only in phase 1 and BRG 19 had activity only in phase 2. E122 had no significant activity in either phase. In the hot plate test only E139 had antinociceptive activity. Administration of either bicuculline, a GABA_A_ receptor antagonist, or CGP 35348, a GABA_B_ receptor antagonist, blocked the antinociceptive activity of E139. In conclusion our results indicate that E139 has antinociceptive activity in the formalin and hot plate tests that are dependent on GABA receptors.

Enaminones are enamines of β-dicarbonyl compounds, whose chemistry and activities in models of diseases or disorders, principally seizures, have been reviewed before^1^,^2^,^3^. Enamines are unstable in aqueous solutions whereas, enaminones are chemically stable. Enaminones are formed by a reaction between a primary amine and a β-dicarbonyl compound. They have been used as intermediates or building blocks in synthetic and medicinal chemistry^1^,^2^,^3^ but they also have biological activities. One of the early studies published as an abstract reported analgesic, papaverine-like, and anticonvulsant activities of an enaminone compound^4^. Another early study investigated the hypoglycaemic activities of enaminone compounds and found that they had no hypoglycaemic activity^5^. However, several studies have shown positive results when the anticonvulsant activity of enaminones were investigated^1^,^6^,^7^,^8^,^9^. The anilino enaminones have been reported to have anticonvulsant activity with minimal adverse effects in *in vitro* and *in vivo* studies^7^,^9^,^10^,^11^,^12^. The anilino enaminone methyl 4-(4′-bromophenyl)aminocyclohex-3-en-6-methyl-2-oxo-1-oate (E139) has been utilised to study the mechanism of action of anticonvulsant enaminones. The anticonvulsant effects of E139 have been attributed to suppression of tetrodotoxin (TTX)-sensitive sodium channels, enhancement of extracellular γ-aminobutyric acid (GABA) levels, activation of α2-adrenoceptors and reversible suppression of glutamate-mediated excitatory postsynaptic currents^1^,^7^,^13^. Other enaminone congeners have also been shown to enhance GABA_A_ receptor mediated responses by acting as positive allosteric modulators^14^.

We recently evaluated the activity of E139 in rodent models of paclitaxel-induced neuropathic pain^15^, taking into consideration that all the above molecules modulated by E139 have been shown to be involved in the pathogenesis or are useful for the alleviation of neuropathic pain^16^,^17^,^18^,^19^,^20^,^21^,^22^,^23^. E139 attenuated paclitaxel-induced neuropathic pain in mice and rats. Moreover, it had antinociceptive activity in naïve mice, in the hot plate test^15^. This suggested that enaminones might be a new class of analgesics, which will be an important addition taking into consideration that there are various types of pain not sufficiently managed with the currently available analgesics. However, there are no reports of studies on the antinociceptive effects of other enaminones. Thus, the objective of this study was to screen a series of enaminones with known effects on neural tissue and mechanism of action on a pain model to determine if some analogues have potential utility in managing pain. We evaluated the antinociceptive effects of five enaminone compounds in mice using the formalin and hot plate tests. The formalin test is used to evaluate persistent nociception caused by peripheral tissue injury and inflammation and is considered as one of the most valid models of clinical acute pain^24^,^25^ and used for evaluation of analgesic activity of potential analgesic compounds^26^,^27^,^28^.

## Results

### Chemistry

The chemical structures of the five enaminone compounds (BRG19, E121, E122, E138 and E139) were completely characterized by spectral and elemental analysis. The chemical structures and C log P data for the five enaminones are shown in Table 1.

### Effects of indomethacin, tiagabine and enaminones in the formalin test

**Figure 1:**
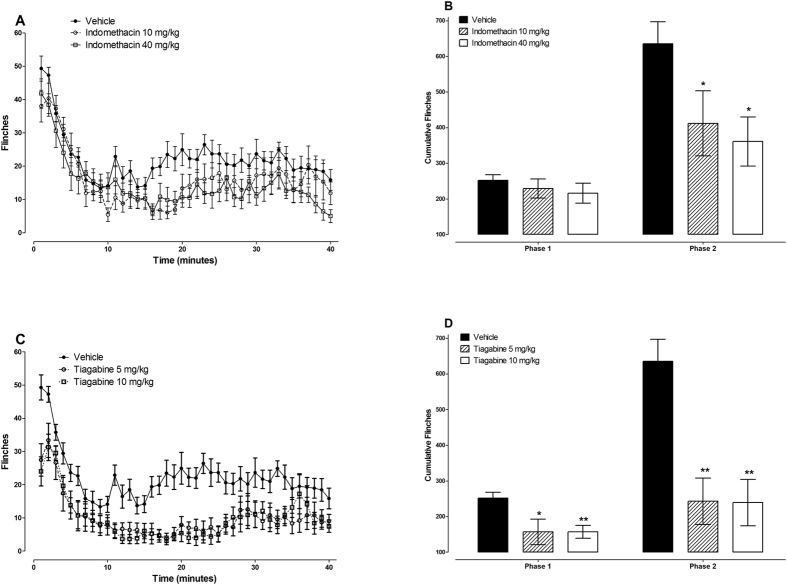
Antinociceptive effects of indomethacin and tiagabine in the formalin test in BALB/c mice. Effects of indomethacin (**A,B**) and tiagabine (**C,D**) on paw flinches induced by injection of 5% formalin s.c. on the paw dorsum measured using an automated flinch detection system from 1 to 40 minutes. (**A,C**) Time course of flinches, (**B,D**) Cumulative flinches phase1 (1–9 minutes) and phase 2 (10–40 minutes) (n = 8–14 per group). *P < 0.05 and **P < 0.01 compared to vehicle-treated mice.

The effects of indomethacin, in the formalin-induced nociception were evaluated as a positive control for a drug with effects on inflammatory nociception and those of tiagabine were evaluated because it increases extracellular levels of GABA in the brain, one of the mechanisms of action of some anticonvulsant enaminones such as E139^7^. Indomethacin is a nonsteroidal anti-inflammatory drug (NSAID), which has been shown to inhibit only the inflammatory phase (phase 2) of the formalin test^26^. On the other hand, tiagabine has been reported to inhibit both phases (phase 1 and 2) of the formalin test^29^. The administration of indomethacin 10 and 40 mg/kg had no significant effect on the phase 1 cumulative flinches in the formalin test (p > 0.05) but significantly reduced phase 2 cumulative flinches from 635 ± 62 to 412 ± 91 and 361 ± 69, respectively (p < 0.05 for both doses; n = 9 for indomethacin 10 mg/kg and 10 for indomethacin 40 mg/kg; Fig. 1A,B). The reductions in phase 2 cumulative flinches caused by indomethacin 10 and 40 mg/kg were 35% and 43%, respectively. The administration of tiagabine 5 and 10 mg/kg significantly reduced both phase 1 and 2 cumulative flinches from 252 ± 16 to 157 ± 36 and 635 ± 62 to 243 ± 65, respectively for 5 mg/kg (p < 0.05 and p < 0.01, respectively; n = 9) and from 252 ± 16 to 157 ± 18 and 635 ± 62 to 239 ± 65, respectively for 10 mg/kg (p < 0.01 for both phases, respectively; n = 8); Fig. 1C,D). Both doses of tiagabine (5 and 10 mg/kg) reduced the cumulative flinches in phase 1 and phase 2 by, 38% and 62%, respectively.

**Figure 2:**
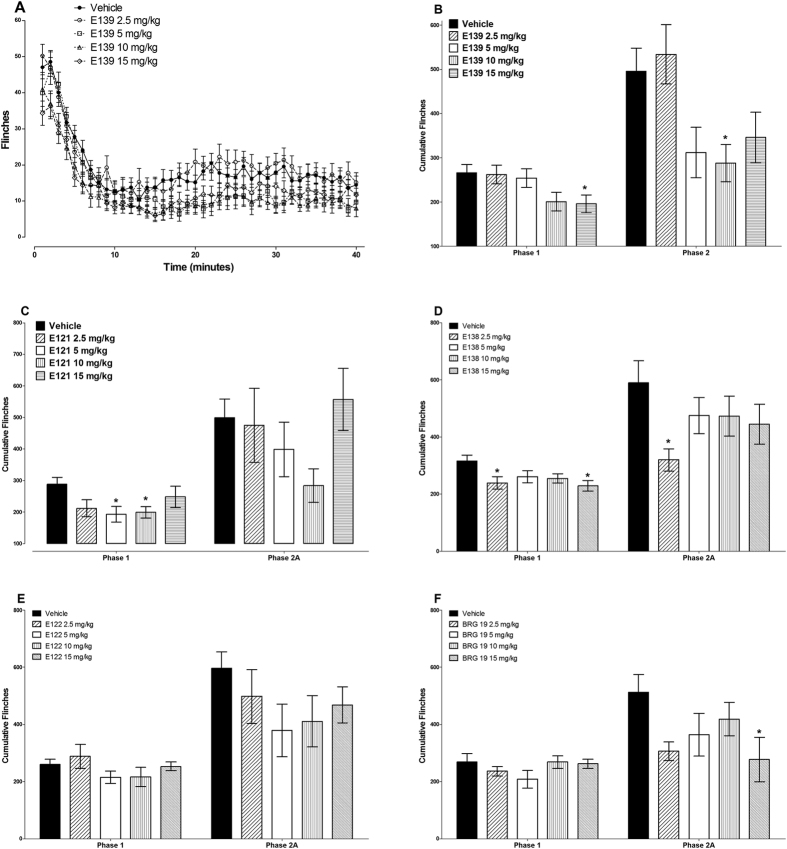
Antinociceptive effects of enaminones in the formalin test in BALB/c mice. Time course of flinches in animals treated with vehicle or different doses of E139 (**A**). Effects of E139 (**B**), E121 (**C**), E138 (**D**), E122 (**E**), and BRG 19 (**F**) on phase1 (1–9 minutes) and phase 2 (10–40 minutes) cumulative paw flinches induced by injection of 5% formalin s.c. on the paw dorsum measured using an automated flinch detection system (n = 8–20 per group). *P < 0.05 compared to vehicle-treated mice.

The administration of E139 significantly reduced both phase 1 and 2 cumulative flinches. Only 15 mg/kg of E139 significantly reduced phase 1 cumulative flinches from 266 ± 19 to 196 ± 23 (p < 0.05), whereas the lower doses did not (p > 0.05, n = 16–23; Fig. 2A,B). On the other hand 10 mg/kg of E139 significantly reduced phase 2 cumulative flinches from 496 ± 52 to 288 ± 42, respectively (p < 0.05), whereas the other doses, did not (p > 0.05, n = 16–23; Fig. 2A,B). The reduction in phase 1 cumulative flinches caused by E139 15 mg/kg was 27%, whereas that produced by 10 mg/kg in phase 2 was 42%.

The administration of E121 significantly reduced only phase 1 cumulative flinches. Two doses of E121 (5 and 10 mg/kg) used significantly reduced phase 1 cumulative flinches from 288 ± 22 to 193 ± 25 and 199 ± 18, respectively (p < 0.05 for all doses; n = 8–9). The percent reductions in phase 1 cumulative flinches caused by E121 5 and 10 mg/kg were 33% and 31%, respectively.

The administration of E138 significantly reduced both phase 1 and 2 cumulative flinches. Two doses 2.5 and 15 mg/kg of E138 significantly reduced phase 1 cumulative flinches from 316 ± 20 to 239 ± 22 and 229 ± 19, respectively (p < 0.05 for all doses; n = 12–14; Fig. 2D). On the other hand 2.5 mg/kg of E138 significantly reduced phase 2 cumulative flinches from 590 ± 77 to 320 ± 39 (p < 0.05), whereas the other doses, did not (p > 0.05, n = 12–13; Fig. 2D). The reduction in phase 1 cumulative flinches caused by E138 2.5 and 15 mg/kg were 24% and 28%, respectively, whereas that produced by 2.5 mg/kg in phase 2 was 46%.

The administration of E122 did not produce any significant changes in the cumulative flinches either in phase 1 or 2 (p > 0.05; n = 10–15; Fig. 2E).

The administration of BRG19 significantly reduced only phase 2 cumulative flinches. One dose of BRG19 (15 mg/kg) used significantly reduced phase 2 cumulative flinches from 513 ± 61 to 277 ± 78 (p < 0.05; n = 8). The percent reduction in phase 2 cumulative flinches caused by BRG19 15 mg/kg was 46%.

### Effects of enaminones and GABA receptor antagonists in the hot plate test

**Figure 3:**
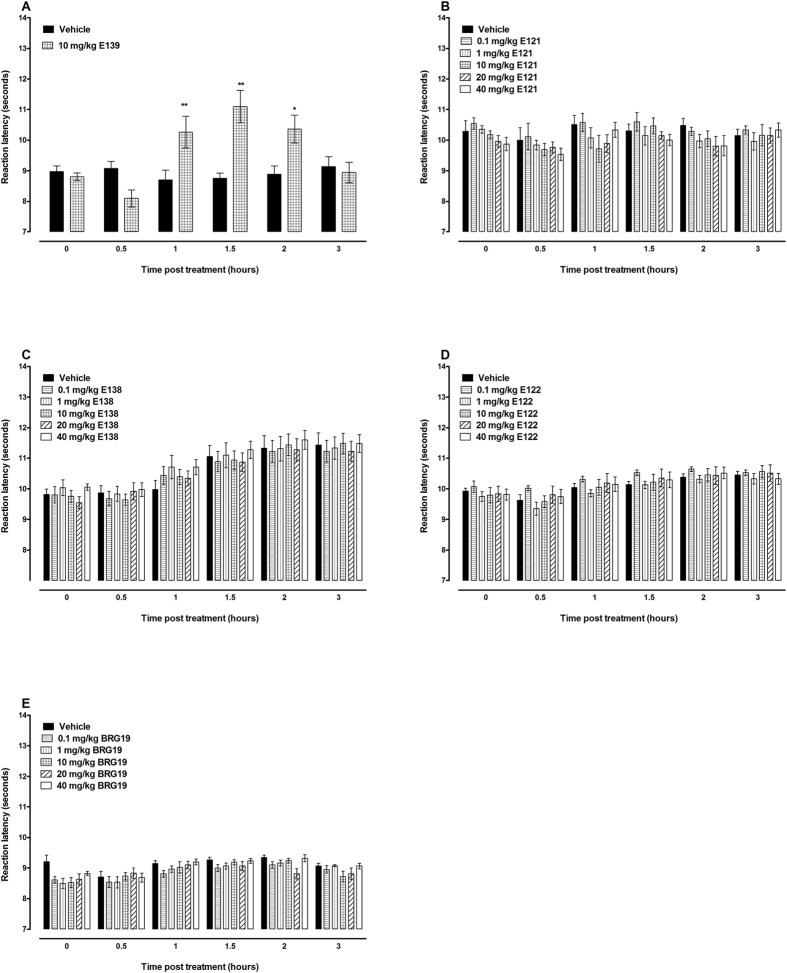
Antinociceptive effects of enaminones in the hot plate test in BALB/c mice. Effects of E139 (**A**), E121 (**B**), E138 (**C**), E122 (**D**) and BRG 19 (**E**) on reaction latency of mice to the hot plate (55 ± 1 °C) at different times after administration (n = 8–12 per group). *P < 0.05 and **P < 0.01 compared to vehicle-treated mice.

**Figure 4:**
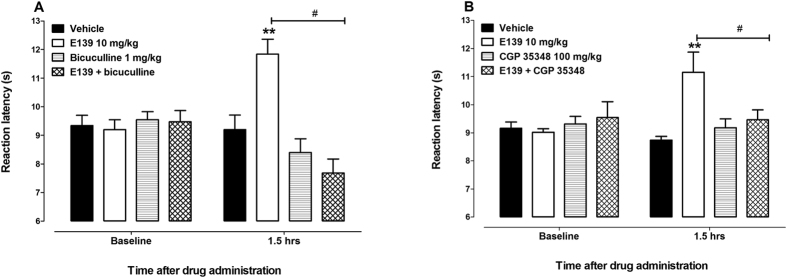
GABA receptor antagonists block the antinociceptive effects of E139 in the hot plate test in BALB/c mice. Effects of (**A**) a GABA_A_ receptor antagonist bicuculline and (**B**) a GABA_B_ receptor antagonist CGP 35348 hydrate on the antinociceptive effects of E139 in BALB/c mice (n = 8) at 1.5 h after administration in the hot plate test. **p < 0.01 compared to mice treated with vehicle and ^#^p < 0.05 and ^##^p < 0.01 to mice treated with E139 at the same time point after treatment.

In the hot plate test, mice treated with E139 10 mg/kg had reaction latency times significantly higher than vehicle-treated animals as we previously described^15^ (p < 0.05; n = 12, Fig. 3A), however E121, E122, E138 and BRG19 (0.1 to 40 mg/kg) had reaction latency times similar to vehicle-only-treated control animals (p > 0.05; n = 8–12; Fig. 3B–F). Baseline values of mice treated with vehicle, E139, bicuculline, which is a GABA_A_ receptor antagonist, CGP 35348, which is a GABA_B_ receptor antagonist, and mice pretreated with either bicuculline or CGP 35348 before treatment with E139 10 mg/kg were similar (p > 0.05; n = 8, Fig. 4). Mice that were treated with bicuculline a GABA_A_ receptor antagonist, had reaction latency times similar to vehicle-only-treated control animals (p > 0.05; n = 8, Fig. 4A) at 1.5 hours after drug administration (a time point when E139 had peak effect, as described previously^15^). Mice pretreated with bicuculline before treatment with E139 10 mg/kg had reaction latency times similar to vehicle-only-treated control animals (p > 0.05; n = 8, Fig. 4A), whereas those treated with E139 alone had higher reaction latency (p < 0.01; n = 8, Fig. 4A) at 1.5 hours after drug administration. Mice pretreated with bicuculline before treatment with E139 10 mg/kg had reaction latency times lower than E139-only-treated animals (p < 0.05; n = 8, Fig. 4A) but similar to bicuculline-only- treated animals (p > 0.05; n = 8, Fig. 4A) at 1.5 hours after drug administration. Mice that were treated with CGP 35348, a GABA_B_ receptor antagonist, had reaction latency times similar to vehicle-only-treated control animals (p > 0.05; n = 8, Fig. 4B) at 1.5 hours after drug administration. Mice pretreated with CGP 35348 before treatment with E139 10 mg/kg had reaction latency times similar to vehicle-only-treated control animals (p > 0.05; n = 8, Fig. 4B), whereas those treated with E139 alone had higher reaction latency (p < 0.01; n = 8, Fig. 4B) at 1.5 hours after drug administration. Mice pretreated with CGP 35348 before treatment with E139 10 mg/kg had reaction latency times lower than E139-only-treated animals (p < 0.05; n = 8, Fig. 4B) but similar to CGP 35348-only-treated animals (p > 0.05; n = 8, Fig. 4B) at 1.5 hours after drug administration. Thus, blocking GABA_A_ or GABA_B_ receptors had similar antagonistic effects on the antinociceptive effects of E139 in the hot plate test.

## Discussion

Recently, we reported that E139, an anticonvulsant anilino enaminone compound, has antinociceptive activity in the hot plate test^15^. Our current study shows that E139 and E138 have antinociceptive activity in both phases of the formalin test, whereas the other enaminones tested (E121, E122 and BRG 19) were active in one phase only or did not have any activity at all. In the hot plate test, only E139 had antinociceptive activity, which was blocked by both GABA_A_ and GABA_B_ receptor antagonists.

Of the five anilino enaminones evaluated only E139 and E138 had significant antinociceptive activity in both phase 1 and 2 of the formalin test. Phase 1 is considered to be due to direct activation of peripheral C-fibres by the irritant (formalin) and thus considered neurogenic, whilst the second phase is considered to be due to inflammatory nociception^28^,^30^. Our results suggest E139 and E138 might have similar activity to drugs that enhance the extracellular levels of GABA such as GABA transporter inhibitors, which inhibit both phases of the formalin test^29^, but not indomethacin, which inhibits only the inflammatory phase^26^. The chemical structure of E139 and E138 has the enaminone system (NH-C=C-C=O), ester and halogen functional group. Specifically, E139 has a methyl ester with bromophenyl group and E138 has a methyl ester with chlorophenyl group. The enantiomers of E139 and E138 existed as racemic mixtures in the compounds. The C log P value for the most active enaminones were 4.06 for E139 and 3.91 for E138. The range of C log P values for all five enaminones was 3.87 to 4.59. There was no correlation between C log P values and antinociceptive activity of the five enaminones evaluated in this study. Therefore, we could not generalize that increase in C log P values of enaminones necessarily increased their antinociceptive activity. This observation was also made with other enaminones when we attempted to correlate C log P values with anticonvulsant activity^10^,^31^. We found no correlation between C log P values and anticonvulsant activity of enaminones. However, with very similar chemical features in the structures of the five enaminones, in this study, it appears that C log P value of about 4.00 was optimum for antinociceptive activity of the enaminone compounds. Even with two strikingly similar enaminones E122 and E139 differing only in the ethyl ester in E122 as compared to the methyl ester in E139, the one with C log P value of 4.59 (E122) was inactive in the hot plate test, while the analog with C log P value of 4.06 (E139) provided antinociceptive effect in the hot plate test. Whereas E139 was active in the formalin test, E122 had no significant activity in the formalin test because it was more lipophilic that E139. The enaminones with much lower or higher C log P values than 4.00 were generally less active. In comparing the activity of E139, E138, and E121, it was observed that E138 and E121 that had chlorophenylamino moiety in their chemical structures exhibited activity only in the formalin test. The difference between the enaminones active in both phases and those active in only one phase or not active in the formalin test is the presence of a methyl ester group (for E139 and E138) versus an ethyl ester group (for BRG19, E121 and E122). The structure-activity relationship (SAR) between E139 and E138 is that a bromo group is required for antinociceptive activity in the hot plate test, if there exists a methyl ester rather than an ethyl ester in the enaminone compound that has a C log P value of about 4.00. Hence BRG19, E121, E122, and E138 were not as active as E139 in the hot plate test. The C log P values indicate the ability of the enaminones to cross membranes and the blood brain barrier and to be distributed throughout the body of the experimental animal. An optimum C log P value of 4.00 of the enaminone was necessary for antinociceptive effect in the hot plate test. There has to be a balance between membrane permeability and the increase in C log P values of medicinal compounds to have the desired pharmacological effect^31^.

One of the mechanisms of action of E139 as an anticonvulsant is the enhancement of extracellular GABA levels in the brain^1^,^7^,^11^. The evidence for an indirect action of E139 was published by Kombian *et al.* in 2005^7^. In that study, the effect of E139 on synaptic responses were blocked by a GABA_B_ receptor antagonist but not a GABA_A_ chloride channel blocker and occluded by a GABA reuptake blocker and a GABA transaminase enzyme inhibitor^7^. Furthermore, a previous report by Mulzac and Scott^32^ showed that the enaminone pharmacophore (compound ADD 196022) did not affect tritriated GABA (^3^H-GABA) binding. This would suggest that direct interaction with a GABA binding site is unlikely for E139. Thus, the mechanism of action of E139 was adduced as indirect possibly via GABA-T inhibition or GABA reuptake inhibition. Drugs that increase extracellular GABA levels in the brain have been shown to have antinociceptive activity in both the formalin and hot plate tests^29^,^33^,^34^. On the other hand, NSAIDs and anti-inflammatory drugs have antinociceptive effects in the formalin test but not in the hot plate test^35^,^36^. E139 had antinociceptive activity in the hot plate test similar to what we previously described^15^, suggesting that E139 has activity similar to drugs that enhance GABA levels. The antinociceptive activity of E139 in the hot plate test was blocked by a GABA_A_ receptor antagonist, bicuculline, and a GABA_B_ receptor antagonist, CGP 35348, the latter similar to what has been described for tiagabine previously^33^, further confirming that the antinociceptive effects of E139 are partly due to the activation of both GABA receptors, possibly by raising the levels of endogenous GABA. Both GABA_A_ and GABA_B_ receptors are involved in regulating pain sensation and agonists of either receptor have antinociceptive activities^37^.

In conclusion, our results show that some but not all anilino enaminones have antinociceptive activities. The active anilino enaminone, E139, has antinociceptive activities in both phases of the formalin test and in the hot plate test that are dependent on activity of both GABA_A_ and GABA_B_ receptors. The antinociceptive activity of E139 is partly due to modulation of the GABAergic system. Thus this anilino enaminone may serve as lead compound for further research and development into novel analgesic agents.

## Methods

### Synthesis of enaminones

The five enaminones BRG19, E121, E122, E138, and E139 were resynthesized and characterized by methods that were previously reported^6^,^8^. In brief, the starting diketo compound was dissolved in absolute ethanol and added to a solution of the corresponding amino compound in absolute ethanol. The reaction mixture was refluxed for 8–10 hours, cooled to room temperature, and evaporated using a rotor vaporator. The crude product was recrystallized from a suitable organic solvent, or solvent-mixture. Spectral and elemental analysis confirmed the chemical structures of the enaminone compounds. The analytical samples of the five enaminones had the following characteristics: BRG 19 had melting point of 150–152 °C with molecular weight of 296.36; E121 had melting point of 161–163 ^o^C with molecular weight of 312.81; E122 had melting point of 151–154 °C with molecular weight of 357.26; E138 had melting point of 178–180 °C with molecular weight of 298.79; and E139 had melting point of 188–190 °C with molecular weight of 343.24.

### C log P determination

The calculated partition coefficient (C log P) values for BRG19, E121, E122, E138, and E139 were determined by using the ChemBioDraw Ultra 14 Suite (Computer software by PerkinElmer). Where the enaminone compound had one or more chiral centers, the enantiomers existed together as a racemic mixture. The C log P values were determined for the racemic mixtures^10^,^31^.

### Animals

Female BALB/c mice (8 to 12 weeks old; 20–30 g; n = 625) used in this study were kept in temperature controlled (24 ± 1 °C) rooms with food and water given *ad libitum*. The animals were supplied by the Animal Resources Center (ARC) at the Health Sciences Center (HSC), Kuwait University, Kuwait. All experiments were performed during the same period of the day (8:00 AM to 4:00 PM) to exclude diurnal variations in pharmacological effects. The animals were handled in compliance with European Communities Council Directive 86/609 for the care of laboratory animals and ethical guidelines for research in experimental pain with conscious animals^38^. All methods were carried out in accordance with the approved guidelines and regulations of the HSC Ethical Committee for the use of Laboratory Animals in Teaching and in Research, Kuwait University. All procedures were approved by the Ethical Committee for the use of Laboratory Animals in Teaching and Research, HSC, Kuwait University.

### Drugs and drug administration

Indomethacin (Sigma-Aldrich, St Louis, MO, USA) was dissolved in phosphate buffered saline (PBS); tiagabine, bicuculline and CGP 35348 hydrate (Sigma-Aldrich, St Louis, MO, USA) in normal saline (NaCl 0.9%) and enaminones (resynthesized in-house^6^,^9^) in peanut oil. The drugs were freshly prepared before administration and administered intraperitoneally (i.p.) to mice at a volume of 10 ml/kg body mass. Indomethacin and tiagabine were administered to mice 1 hour before subcutaneous (s.c.) administration of formalin (5%; 20 μl). Enaminones were administered 1.5 hours before administration of formalin, taking into consideration the time the enaminone E139 produced significant antinociceptive effect in the hot plate test^15^. Bicuculline, a GABAA receptor antagonist, and CGP 35348 hydrate, a GABA_B_ receptor antagonist, were administered 15 minutes before the administration of E139 for the hot plate test.

### Formalin test

An automated formalin test (automated nociception analyzer, ANA) developed by Yaksh *et al.*^39^, was used to evaluate chemical nociception as described previously^40^. Small metal bands were placed around the base of mice left hind paw and fixed in place with cyanoacrylate glue. The mice were placed in a cylindrical test chamber at least 1 hour before administration of formalin into the paw dorsum. After formalin injection, mice were returned to the chamber and flinches counted for 40 min with an automated device as described before^39^,^40^. When formalin is injected into the paw it induces nociception in mice, which causes the mice to flinch and raise the paw to lick it. Movement of the metal band on the mouse’s paw (during flinching) alters the electromagnetic field. The resulting signal was fed to a computer that uses the response amplitude and duration to separate flinches from normal locomotor activity. Formalin induces nociception in mice in a biphasic manner. The phases of the formalin test were defined as phase 1 also known as early phase (neurogenic nociception) from 1–9 minutes and phase 2 also known as late phase (inflammatory nociception) from 10–40 minutes. Cumulative flinches for each phase, i.e. sum of flinches from 1 to 9 minutes for phase 1 and from 10 to 40 minutes for phase 2, were compared between the vehicle-treated and the drug-treated animals.

### Hot plate test

Reaction latencies to hot plate test were measured before (baseline latency) and after drug administration. Briefly, mice were placed on a hot plate (Panlab SL, Barcelona, Spain) with the temperature adjusted to 55 ± 1 °C. The time to the first sign of nociception, paw licking, flinching or jump response was recorded and the animal immediately removed from the hot plate. A cut-off period of 20 seconds was maintained to avoid damage to the paws.

### Data and statistical analyses

The software GraphPad Prism version 5.00 (GraphPad Software Inc., USA) was used for plotting graphs, data and statistical analyses. Statistical analyses were performed using unpaired Student’s t test when only one dose was used, one-way analysis of variance (ANOVA) followed by Newman-Keuls Multiple Comparison Test for multiple dose effect for each compound, two way ANOVA followed by Bonferroni post-tests for dose effect over time. The differences were considered significant at p < 0.05. The results in the text and figures are expressed as the means ± S.E.M.

## Additional Information

**How to cite this article**: Masocha, W. *et al.* Evaluation of the antinociceptive activities of enaminone compounds on the formalin and hot plate tests in mice. *Sci. Rep.* **6**, 21582; doi: 10.1038/srep21582 (2016).

## Figures and Tables

**Table 1:**
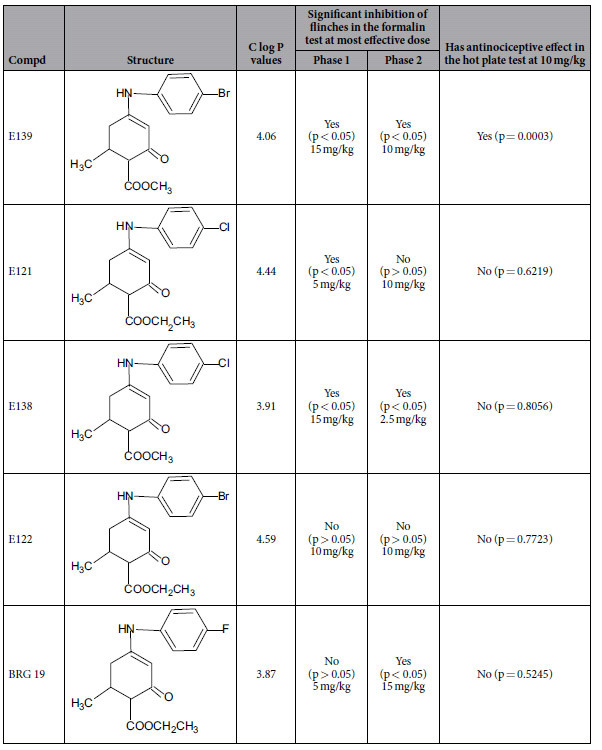
Structures of evaluated enaminones, C log P values, inhibition of flinches by enaminones in the formalin test and antinociceptive effect in the hot plate test after 1.5 h at most effective dose.
